# 557 Introduction of a Pediatric Burn Education Program in an Adult Hospital

**DOI:** 10.1093/jbcr/irac012.185

**Published:** 2022-03-23

**Authors:** Emily Snyder

**Affiliations:** Medical City Plano, Plano, Texas

## Abstract

**Introduction:**

At a newly developed burn unit, the program decided to expand further and start admitting pediatric patients. While there are many working parts to this endeavor, we will primarily address staff preparedness. Prior to the introduction of a burn education course entitled *Burns in the Pediatric Population*, only a handful of nurses had received any hospital-based education for caring for pediatric patients. A previous pediatric course had been taught, however, this course focused primarily on illnesses of childhood. Staff had voiced on many occasions that they felt the education they received was not adequate and felt uncomfortable taking care of pediatric burn patients.

**Methods:**

All Burn Intensive Care (BICU) nurses, regardless of having received the prior pediatric education, were required to take *Burns in the Pediatric Population* (n=42). The course content was based on the Burn Nurse Competencies. The course consisted of didactic lectures and hands-on sessions. Each participant was required to take a pre-test before the class and a post-test at the conclusion. The test included knowledge-based questions and self-rated confidence level questions. In addition, each participant was sent a survey three months after the completion of the class to evaluate their knowledge and confidence level.

**Results:**

At the conclusion of the class, the average test score went from 49.3% to 92.7%. Both the pre-test and post-test had each nurse evaluate their own confidence level for caring for a pediatric patient. Initially, 19.5% of the nurses stated that they had no confidence in caring for a pediatric patient. At the conclusion of the class, all nurses expressed some confidence with caring for a pediatric patient, with the majority, 72.7%, stating they had moderate or high confidence.

The return rate of the three-month evaluation was 81% (n=34). The knowledge-based test had an average score of 71%. 30.3% of the staff stated that their confidence in caring for a pediatric patient increased, 54.5% stated their confidence level remained the same, and 15.1% of those returning the survey stated that their confidence level decreased in the three month time period.

**Conclusions:**

The results from the three-month survey have been utilized to edit and make our pediatric mock codes and course more specific to the needs of the bedside nurses. In addition, we are planning to increase the frequency and the level of participation in our pediatric mock codes. All Burn ICU nurses will need to participate in a pediatric mock code on a semi-annual basis. In addition, there will be a section that is added to each Burn ICU nurse’s annual competency specifically covering pediatric burns.

